# Hope pluralism in antenatal palliative care

**DOI:** 10.1136/jme-2024-110120

**Published:** 2024-10-14

**Authors:** Sophie Bertaud, Mehrunisha Suleman, Dominic Wilkinson

**Affiliations:** 1 Oxford Uehiro Centre for Practical Ethics, University of Oxford, Oxford, UK; 2 Louis Dundas Centre for Children's Palliative Care, Great Ormond Street Hospital for Children NHS Foundation Trust, London, UK; 3 Ethox Centre, University of Oxford, Oxford, UK; 4 Gulf University for Science and Technology, Hawally, Kuwait; 5 John Radcliffe Hospital, Oxford, UK

**Keywords:** Palliative Care, Pediatrics, Fetus, Obstetrics, Prenatal Diagnosis

## Abstract

When parents face the distressing news during pregnancy that their baby is affected by a serious medical condition that will likely lead to the baby’s death before or soon after birth, they experience a range of complex emotions. Perhaps paradoxically, one common response is that of hope. Navigating such hope in antenatal interactions with parents can be difficult for healthcare professionals. That can stem from a desire to accurately communicate prognostic information and a fear of conveying ‘false hope’ to families. In this paper, we examine the role that hope plays when parents and healthcare professionals are grappling with a confirmed antenatal diagnosis of a life-limiting condition. We assess what it means to hope in this context and consider the different types of hopes held by both parents and healthcare professionals as well as why hopeful thinking might be helpful and not harmful. We propose ‘hope pluralism’ as a concept that might allow healthcare professionals to accommodate a multitude of parental and professional hopes, even where these conflict. Finally, we offer some practical suggestions for how professionals should evaluate and respond to hope in situations that might (from the outside) appear hopeless.

Hope is not a form of guarantee; it’s a form of energy, and very frequently that energy is strongest in circumstances that are very dark. John Berger

##  Case example^i^

*Christine and her husband James are 28 weeks pregnant with their first child, a male fetus who they have named Ewan. Ewan was diagnosed with anencephaly (a condition in which major portions of the brain, skull and scalp are absent) at 12 weeks’ gestation. The fetal medicine team have told Christine and James that Ewan is unlikely to survive for more than a few hours after he is born and have suggested they meet with the paediatric palliative care team to discuss plans for Ewan’s care after birth. Christine and James are reluctant to meet the palliative care team as they are still hopeful that the scans will turn out to be mistaken and that Ewan will be born healthy. The fetal medicine team are concerned that Christine and James do not grasp the severity of the situation and are in denial.*

### Introduction

When faced with a devastating diagnosis such as anencephaly, parents respond with grief, disbelief, isolation, anger and adaptation, but also, frequently, with hope.^1^,^3^ What does it mean to hold ‘hope’ after an antenatal diagnosis of a severe and potentially life-limiting condition? How ought professionals to respond to hope in this context?

The aim of this paper is to respond to the latter ethical challenge in clinical practice. We will do so by first examining what it means to hope in this context, considering why hope might be helpful for parents like James and Christine despite the apparently bleak outlook, and outlining some of the potential challenges of hope that professionals deem to be inappropriate. We propose an approach of ‘hope pluralism’ and offer some practical suggestions for how this might be applied in clinical care. This paper offers a conceptual ethical analysis derived from relevant empirical and ethical literature and informed by the clinical experience of the authors. However, it is important to acknowledge that this is not an empirical or sociological report nor a psychological model of parental adjustment.

First, it will be useful to clarify what we mean by hope. There are different potential definitions and theoretical accounts. Most centre around the idea of an expectation or an anticipation of a desired positive outcome or an improved state in the future.^4 5^

Based on data from elderly patients with cancer, Dufault and Martocchio^6^ define hope as ‘a multidimensional dynamic life force characterized by confident yet uncertain expectation of achieving a future good which to the hoping person is realistically possible and personally significant’. Snyder^7^ defines it as ‘the perceived capability to derive pathways to desired goals, and motivate oneself via agency thinking to use those pathways’. Common elements of these definitions include:

*Hope*: a positive attitude towards a desired but uncertain future outcome.

### Holding hope in uncertainty

On these standard accounts, hope necessarily relates to the potential that the future positive outcome will be achieved.^8^ We can only hope for a future state of affairs if we believe the probability of that future state of affairs to be between 0 and 1.^9^ To want something to happen or be true, we usually need to have a good reason to think that it might. Where there is a non-zero possibility, it is reasonable, and indeed often encouraged, to be hopeful that a desired outcome will be realised. This has relevance for antenatal consultations where there is frequently considerable uncertainty when a potentially life-limiting condition is diagnosed.^10^,^12^

There may be several layers of uncertainty that parents and healthcare professionals are navigating together:

- Uncertainty about the diagnosis itself due to the limitations of what prenatal imaging or testing can achieve. An example would be complex congenital heart disease where the limitations of diagnostic technologies are such that postnatal imaging would be needed to confirm whether beneficial surgical intervention is possible.^13 14^
- Uncertainty due to the very nature of the diagnosis itself, where a condition may evolve over time and where the natural course is inherently unpredictable. An antenatal diagnosis of severe kidney problems would be an example of this; the condition requires monitoring over time, there may be a risk of intrauterine death and the outcome is highly variable.^15^ Initial information from scans before birth is often insufficient to predict postnatal outcome.
- Even with a definitive prenatal diagnosis, there may be considerable uncertainty about the baby’s expected length of survival. An example would be the severe brain malformation, holoprosencephaly, where even with detailed prenatal neuroimaging, it can be still difficult to predict the baby’s life expectancy.^16^

Where the outcome is particularly uncertain, healthcare professionals would usually want to encourage parents to remain hopeful and are unlikely to find this difficult or jarring. For the purposes of this paper, we will begin by considering those cases where there is a high degree of certainty: where the diagnosis has been confirmed and the chance of the baby surviving beyond the first year of life is small. Examples of conditions in this category would include an antenatal diagnosis of Trisomy 13 or Trisomy 18 (severe chromosomal problems), renal agenesis (congenital absence of kidneys) or anencephaly (such as in our case example). It is hope in those cases that, we suggest, is most likely lead to ethical discomfort. In the final part of the paper, we will return to consider whether insights offered here might also help with cases where prognostic uncertainty exists.

### Why might hope be helpful? (despite certain prognosis)

Hope has been extensively examined in the palliative care literature, particularly as it relates to adults dying from a terminal illness. Hope is recognised as a positive coping mechanism and a source of energy.^17^ There is evidence that specific psychosocial interventions can be effective in increasing patient hope.^18^ For parents caring for a child with a chronic condition, hope can act as a resilience factor; mothers reporting higher levels of hope exhibit less distress under conditions of high caregiver stress.^19^ Healthcare professionals play a key role in facilitating hopeful thinking in their patients, in helping them identify important goals and increasing their sense of agency.^20^

We might assume that a prospective parent who receives the diagnosis of a life-limiting condition in their baby during pregnancy has their hopes all but obliterated by such news. However, case studies suggest that for these parents too, hope can function as a coping strategy and a source of strength.^21^ Even faced with clinical information that denies the prospect of cure or long-term survival, parents demonstrate that the process of hoping endures, and parental hopes can usually be elicited by asking them what it is they are hoping for now.^22^ For example, Janvier *et al*^23^ report a number of hopes which appear to be common to parents when they receive a diagnosis of trisomy 13 or 18, such as a hope to meet their child alive, to take their child home, to be a family and to give their child a good life.

In a study of parents of children receiving palliative care, parents who had higher levels of hope were better at transitioning from one set of goals, such as goals focused on cure, to another set of goals, such as goals focused on comfort, and were more likely to make decisions to limit interventions.^24^ Hill and Feudtner^5^ argue that rather than being experienced as a form of denial, hopeful thinking may therefore help parents to ‘regoal’ or to shift from one set of goals to another over time.

### Inappropriate hope

While it may sometimes clearly be helpful to support parental hope in the antenatal period, there are also occasions where healthcare professionals deem parental hope to be inappropriate. This is usually when the probability of the hoped-for outcome being achieved is so small that it is felt to be almost impossible; the hope is not attached to a realistic assessment of the potential outcomes. For the purposes of this discussion, we will take inappropriate hope to be hope that is, according to a professional assessment, not tracking with, or responding to, the relevant facts.

As in the case example of Christine and James, it is not unusual in our experience for parents to hold a seemingly irrational belief that the diagnosis is mistaken and that when the baby is born, they will be perfectly healthy. They may conceal this hope from professionals, but when probed many parents will admit to holding this as a hope. This is sometimes met with disapproval, and at worse derision, from healthcare professionals who may assume that parents have simply not understood the information presented to them or are acting from a place of denial. Yet it is possible to hope for something that is extremely unlikely, and the anticipation of a positive outcome in the future need not be founded on concrete, real-world evidence.^4^

The presence of hope in the clinical consultation can act as both an asset and a challenge for professionals.^25^ Professionals may simply struggle to reconcile the fact that hope which is generally ‘future-orientated’ is seemingly incompatible with the obvious reality that palliative care patients have a necessarily limited future ahead of them.^26^ Paediatricians have described a tension between the emotional aspect of hope and the intellectual understanding of a prognosis and report a professional desire for hopeful parents to ‘acknowledge the possibility of a negative outcome’.^27^

Hope that is felt to be inappropriate sits uncomfortably with healthcare professionals. In response to inappropriate hope, professionals may choose to double down on their efforts to explain the condition and its prognosis, potentially causing further alienation for families.^28^ Or the professionals may themselves withdraw in the face of an emotional response that appears contrary to reason and perhaps therefore beyond the point of rational engagement.

A separate potential concern in the antenatal setting is the clinician’s fear of parents coming back to them after a child is born, with accusations that they had not been adequately counselled about their child’s prognosis. In some cases, parental hope is presented as a fixed belief; seemingly unresponsive to evidence to the contrary. Professionals may fear that parents will not be able to adjust to ‘reality’ when it arrives. As noted by Ruddick,^29^ failed hopes not only disappoint; they often cause prolonged suffering. So, in the case of Christine and James, professionals may be worried that when Ewan is born his parents will struggle more if they have not begun to assimilate the facts of his condition before birth.

Alternatively, by colluding (even passively) with an inappropriate hope, some argue that healthcare professionals may be inflicting harm, particularly if the hope results in requests for a healthcare intervention (eg, caesarean section or neonatal resuscitation) that deviates from the ‘standard of care’.^30^ Inappropriate hopes may prevent a parent from accessing care that may be important. Christine and James have refused to meet the palliative care team before birth and will therefore miss out on the opportunity to engage in parallel planning for Ewan’s care after birth. For example, this may influence where he can be cared for and preclude the opportunity for a transfer home prior to death.^31^

Inappropriate hope is sometimes out of the realms of what professionals feel they can engage with personally. This may point to fundamental differences in the value or belief systems between parents and the professional. For Christine and James, professionals may simply be struggling to support the parents’ decision to continue with the pregnancy in the face of such a severe prenatal diagnosis; this may jar with their own beliefs about quality of life. Similarly, in many cultures, death is a taboo subject; discussions about death are avoided even for those that are dying because it is believed that such conversations may hasten the dying process.^32 33^ Parental reluctance to engage with discussions around their baby’s death may clash with the cultural expectations of a westernised health system where acknowledging prognostic information is encouraged.^34^

### Hope pluralism

How should healthcare professionals respond when parents articulate hopes that are deemed inappropriate by the medical teams?

One of the challenges to the idea of inappropriate hope is the recognition that appropriateness clearly reflects a value judgement. Articulating such judgements can seem profoundly disrespectful to the parents’ own worldview. It may also seem strikingly insensitive to insist (for parents who face the awful prospect of losing their child), that they must respond emotionally in a particular, sanctioned or approved way.

But holding inappropriate hopes may seem less problematic if we allow for the idea that there is not a single right way of responding to extremely bad news, and that people might simultaneously hold a diversity of hopes. We could encourage an approach that we could call:

 *Hope Pluralism*: a view that multiple, potentially divergent forms of hope can coexist and be valid within a given context. Healthcare professionals should respect and accommodate a range of different hopeful responses, even in the face of bad news.

This approach recognises that different individuals, such as patients, families and healthcare professionals, may have different perspectives, desires and expectations about the future. It is based on the understanding that hope is a deeply personal and subjective experience, and what is considered meaningful hope can differ greatly from person to person. The concept that we are describing here overlaps with the well-described philosophical notion of value pluralism; that is, that there are different sources of moral goodness and that these conflicting values may be equally important.^35^ In our view, hope is not purely an emotion but also necessitates a certain type of valuing. However, to our knowledge, there have been no previous descriptions of a pluralistic approach to hope. Pluralism allows for the complexity and conflict that is part of our moral experience^36^ and in this way is particularly apt for antenatal palliative care, where hopes may naturally coexist and conflict with each other. Value pluralism is also linked to the idea that values are incommensurable; that is, that there may not be a way of objectively comparing them or deciding which course of action is best. In the same way, parental hopes after a life-limiting diagnosis may be in tension without any necessary way of reconciling them. A pluralistic understanding could help professionals to come to terms with parental hope that persists even when news appears grim, or to support parents who carry simultaneous contradictory hopes.

Our vision of hope pluralism is also consonant with *political pluralism*, the idea that within a community there will inevitably be different, strongly held opposing views, but that we should aim for these to peacefully coexist.^37^ For hope, professionals should where possible respect a range of different hopes, including those that they personally do not share. They should expect that in the same circumstance different patients will hold different hopes, and not see this as necessarily problematic.

### Hope in the face of hopelessness

If healthcare professionals try to be hope pluralists, what would that mean for practice in an area like antenatal palliative care? Just as a multitude of hopes might be permitted to exist, our response to hope might also not be singular. Medical professionals should give parents permission to hope for things that may be unlikely and ought to respond in a way that does not necessarily match their personal response to the situation. This is not to say that it would be advisable to always align with parental hope and ignore the evidence or information that professionals have access to. But even if we disagree with another person’s hope, we can potentially come to accept and respect it. Professionals can be responsive to parental hopes and accept hopes that may seem immutable, and which contradict with their own beliefs. This may require professionals to do away with a judgement of parental hopes and to instead prioritise an approach grounded in compassion.

Allowing a space for plurality may help to foster more supportive relationships with families. Hope pluralism would allow for different types of hope to be permitted within the antenatal consultation and allow professionals to tailor their discussion of hope to the individual family in front of them. This might involve an open-minded exploration of what the parents are hoping for, and what hope itself means to them. This may allow conversations to take place that might not have otherwise happened. Parental responses to bad news will also inevitably be informed by cultural and religious factors and healthcare professionals ought to be responsive and tolerant to this. Allowing for hope pluralism may be one first step towards cultural humility^38^ in antenatal consultations.

In our case example, when Christine and James articulated their hope that Ewan’s scans would prove to be mistaken, a professional response which acknowledged and allowed space for this hope to exist could have been beneficial. This may be as simple as stating ‘thank you for telling me that. Whilst I think that is extremely unlikely, I can see why you would hope for that’. It can also help to reassure parents that their hopes are not unusual, by saying something such as ‘you know, a lot of the parents that I see in this sort of situation describe that sort of feeling. It is very natural when faced with this incredibly difficult news to hope that there has been some sort of mistake. Sometimes, holding on to hope, helps people to keep going…’

This may then allow space to discuss whether there is anything else that Christine and James might be hoping for, and an opportunity to explore their priorities for when Ewan arrives. For example, they may simultaneously be holding a hope that Ewan does not suffer when he is born. If their first hope had been supported in the consultation, this second hope might have been more readily addressed.

Furthermore, acknowledging the coexistence of multiple emotional states, such as hope and acceptance, within the antenatal context is crucial. Parents often maintain hope while also acknowledging the reality of their situation, illustrating the intricate nature of emotional experiences in such settings. This complexity is exemplified by Suleman’s^39^ exploration of the Islamic concept of steadfastness (taqwah), which suggests that hope and acceptance can harmonise within a religious framework. This recognition of the simultaneous presence of hope and acceptance echoes experience in paediatric palliative care. In a study of parents of children with relapsed or refractory cancer, many parents hoped for a cure and also reported that they did not believe cure was possible; parents were often able to directly acknowledge this contradiction.^40^ In a collaborative project between bereaved parents and palliative care doctors, Kaye *et al*^41^ argue for a model of hope that validates a parent’s ability to experience hope and prognostic awareness simultaneously.

Following a prenatal diagnosis, parents have reported feeling that their clinicians are overly fatalistic and biased in their presentation of available choices.^42^ Parents may sense that professionals are trying to ‘drum out all of [their] hope’.^43^ Hope pluralism would allow professionals to communicate support to families when they articulate multiple or contradictory hopes.

We have focused on cases of diagnostic/prognostic certainty. However, in many cases of antenatal diagnosis of a life-limiting condition, there will not be such certainty. While hope is less likely to cause a problem in cases where the baby’s outlook remains very uncertain (both parents and professionals are likely to be hoping for the best possible outcome), the concept of hope pluralism may still be pertinent; it will allow professionals to accommodate a range of possible parental responses. For example, one situation in which it may be helpful is if parents seemingly *lack* hope in the case of a very uncertain fetal diagnosis. In this scenario, it may be the professionals who are more hopeful of a positive outcome as compared with the parents (consider, eg, correctable forms of congenital heart disease or brain abnormalities that are often (but not always) associated with normal development). Maintaining a pluralistic approach here might allow professionals to sit with parental responses or choices (such as a termination for medical reasons) that do not align with the professional’s own.

### Potential criticisms

Although we have proposed a pluralistic approach to hope, there are some potential objections:

Some may argue that by adopting hope pluralism professionals may be forced to agree with even the most seemingly irrational hopes presented to them. But hope pluralism need not equate with hope *relativism*. Not all hopes are equal. Some may be more or less apt, and professionals do not need to agree with hopes (eg, for miraculous cure) that appear to have no prospect of realisation. Rather, we suggest that where parents have hopes that differ from those of healthcare professionals, they have a right to have them heard and explored. Even if professionals don’t share their patient’s hopes, they can come to understand and respect why the parents feel that way.^44^

Some healthcare professionals may feel that their role in the antenatal consultation is to communicate accurate prognostic information, but that they are not responsible for what their patients make of that information or how they choose to respond to it. Evaluating, replying to, or even manipulating a parent’s emotional response, hopeful or otherwise, may simply be out of the scope of a doctor’s role. And yet we would argue that caring for patients necessarily involves professionals being receptive and responsive to what patients need.^45 46^ For parents facing the possibility of losing their baby, they may often need us to engage with their hopes. Compassionate antenatal care begins from a place where healthcare professionals are able to apprehend the reality of what parents may be facing, including what they might be hoping for.

The strongest objection to hope pluralism may be those cases where inappropriate hope has the potential to cause harm. If hope pluralism were to oblige healthcare professionals to accommodate hopes which are harmful, that may contradict with their duty of care. Christine and James’ hope that their baby would survive prevented them from accessing palliative care services. However, the goal here should not be to take hopes away from parents, but rather to add hopes in. Professionals should allow space for Christine and James to continue to lean on their hope for Ewan’s survival, but also offering new hopes as extra support, and in this case presenting the palliative care team as a means of facilitating new hopes.

Being supportive of parental hopes that do not track with the medical facts does not mean these hopes must then dictate medical interventions. A medical team can make a professional evaluation that invasive ventilation at birth would not be in a baby’s best interests and decline to perform such an intervention. They can communicate this decision, and their rationale for making it, while still respecting parental hopes for an alternative outcome.

## Conclusions

In the darkest of circumstances, hope endures; and while hope may not be a form of guarantee, it is, as John Berger describes, a vital source of energy that can enable individuals to confront apparent hopelessness.

When a baby is diagnosed with a life-limiting condition during pregnancy, responding to parental hope can feel challenging and uncomfortable for healthcare professionals. But as we have argued in this paper, hope is not merely an emotional response; it is necessarily evaluative. If we are value pluralists, we should expect that attitudinal evaluations (like hope) are multiform rather than singular. But even if we are not value pluralists, there is a strong political pluralist argument for hope pluralism. Professionals should respect and support a range of hopeful responses, even where that diverges from their own preferred response.

We have outlined a concept of hope pluralism that may help professionals to respond to hope in a novel way. We have focused on antenatal palliative care, but this approach may have relevance for other clinical scenarios in which patients and healthcare professionals are together navigating hope in the face of an apparently bleak prognosis.

## Data Availability

Data sharing not applicable as no datasets generated and/or analysed for this study.

## Footnotes

All names are fictional.
